# CONTROL BELIEFS MEDIATED BY SELF-OBJECTIFICATION IN RELATION TO ADULT WOMEN’S SELF-ESTEEM

**DOI:** 10.1093/geroni/igad104.1913

**Published:** 2023-12-21

**Authors:** Aurora Sherman

**Affiliations:** Oregon State University, Corvallis, Oregon, United States

## Abstract

Beliefs about mastery of one’s environment as well as constraints on one’s personal control are predictive of well-being across mid-life and older adulthood. In addition, women’s level of self-objectification is predictive of well-being in early adulthood, but less is known about the role of self-objectification and well-being for mid-life or older women. This study investigated the role of control beliefs (mastery and constraints) and self-objectification in their relationship to the self-esteem of a sample of adult women (rage 48-90, Mean age = 67.83 years). We found that, compared to the mid-life women, older adult women reported higher self-esteem as well as lower constraints and self-objectification, but no age group differences in mastery beliefs. Further, using Hayes’ PROCESS model 4 we show that both mastery and constraints beliefs are mediated in their relationship with women’s self-esteem through self-objectification (R2 = .47 for the model with Mastery and R2= .37 for the model with constraints). Higher mastery predicted lower self-objectification, which, in turn, predicted higher self-esteem, while higher constraints predicted higher self-objectification and lower self-esteem. Additional analyses revealed that age remained a significant independent variable of self-esteem and was not mediated by control beliefs or self-objectification. Results are discussed in light of positive aging and objectification theory.

